# Severe dopaminergic neuron loss in rhesus monkey brain impairs morphine-induced conditioned place preference

**DOI:** 10.3389/fnbeh.2015.00273

**Published:** 2015-10-12

**Authors:** Ting Yan, Joshua Dominic Rizak, Jianhong Wang, Shangchuan Yang, Yuanye Ma, Xintian Hu

**Affiliations:** ^1^Key Laboratory of Animal Models and Human Disease Mechanisms, Kunming Institute of Zoology, Chinese Academy of SciencesKunming, Yunnan, China; ^2^University of Chinese Academy of SciencesBeijing, China; ^3^Kunming Primate Research Center, Kunming Institute of Zoology, Chinese Academy of SciencesKunming, Yunnan, China; ^4^Yunnan Key Laboratory of Primate Biomedical ResearchKunming, Yunnan, China

**Keywords:** dopamine, morphine, reward, place preference, rhesus monkey

## Abstract

It is well known that dopamine (DA) is critical for reward, but the precise role of DA in reward remains uncertain. The aim of this study was to determine what percentage of dopaminergic neurons in the primate brain is required for the expression of conditioned reward by measuring the performance of DA-deficient rhesus monkeys in a morphine-induced conditioned place preference (CPP) paradigm. Animals with mild Parkinsonian symptoms successfully developed and retained a morphine preference that was equivalent to control monkeys. However, these monkeys could not maintain the preference as well as controls when they retained severe Parkinsonian symptoms. On the other hand, monkeys initially in a severe Parkinsonian state developed a preference for morphine, but this preference was weaker than that of the controls. Histological results showed that the loss of dopaminergic neurons in monkeys that had severe Parkinsonian symptoms was about 80% in comparison to the control monkeys. All these data suggest that a severely impaired DA system alters rewarding-seeking behavior in non-human primates.

## Introduction

Research, using diverse methods, has converged on the point that the dopamine (DA) system is fundamentally important to reward related behaviors (Berridge and Robinson, 1998; Hyman, 2005; Palmiter, 2008). It has been well established that drugs producing rewarding effects are dependent on their ability to elevate extracellular DA levels in the mesolimbic DA system (Koob and Volkow, 2010). Morphine, for example, inhibits γ-amino-butyric acid (GABA) neurons in the ventral tegmental area (VTA) through the binding of μ-opioid receptors, which results in the activation of mesolimbic dopaminergic neurons (Nestler, 1992; Jalabert et al., 2011). In fact, animals that self-administer morphine directly into the VTA have been found to display a place preference (Altarifi and Negus, 2011), whereas administration of DA antagonists that block mesolimbic DA transmission inhibits drug reward (Manzanedo et al., 2001).

Despite this ample evidence pointing to an essential role for DA in mediating reward related behaviors (Spanagel and Weiss, 1999; Flagel et al., 2011), the amount of dopaminergic neurons that are needed in reward in mammalian brains remains uncertain. To investigate this important question, the performance of rhesus monkeys in conditioned place preference (CPP) tasks was used as a marker of reward behavior in this study. CPP has commonly been employed in drug-reward experiments in which animals repeatedly receive a drug in distinct chambers of a conditioning room and are subsequently tested for their preference for each chamber (Tzschentke, 2007). Rhesus monkeys were used here because these primates are close relatives of humans and have the ability to exhibit a long-lasting morphine-induced CPP, as demonstrated previously in our laboratory (Wang et al., 2012). These characteristics of the rhesus monkey make it an excellent model system to study whether monkeys can establish and retain a morphine CPP while burdened with impaired DA systems. Inducing different levels of DA neuron loss will establish how much dopaminergic neuron loss is needed for significant differences between DA-deficient and control monkeys.

Dopaminergic neuron loss in the monkey brain was induced in this study by 1-methyl-4-phenyl-1,2,3,6-tetrahydropyridine (MPTP) toxicity. MPTP is a neurotoxin that causes the selective death of dopaminergic neurons and as a result, a decrease in the level of DA through the inhibition of mitochondrial energy metabolism (Morfini et al., 2007). Humans and monkeys receiving MPTP produce the entire triad of Parkinsonian symptoms: bradykinesia, tremor and rigidity (Mounayar et al., 2007). Monkeys in this study were assigned into different groups to develop mild or severe Parkinsonian symptoms by receiving two different MPTP intoxication protocols. The monkeys’ Parkinsonian symptoms were graded to estimate the degrees of impairment to the DA systems in the brain. Then they were tested for morphine CPP. The preference scores among the groups were compared to find out whether different levels of DA system damage lead to different levels of drug preference and to elucidate the precise role of DA in morphine reward.

## Materials and Methods

### Animals and Surgery

This study was carried out in strict accordance with the guidelines for the National Care and Use of Animals (P.R. China) as approved by the Institutional Animal Care and Use Committee (IACUC) of the Kunming Institute of Zoology (approval ID SWYX-2010012). All efforts were made to minimize suffering.

Twelve male rhesus monkeys (*macaca mulatta*; 8–10 years old, 8–11 kg) from the breeding colonies of the Kunming Institute of Zoology were used in this study. These animals were housed under controlled conditions of humidity (60%), temperature (20°C ± 2°C) and light (12:12 h light:dark cycle). All the animals were fed with commercial monkey biscuits twice a day and water was available *ad libitum*. Fruits and vegetables were given to the monkeys once daily.

In accordance with the MPTP intoxication protocols, the 12 monkeys were divided into three groups: group I (*n* = 5, intramuscular MPTP administration), group II (*n* = 3, intracerebroventricular MPTP administration) and the control group (*n* = 4, intramuscular saline administration). Because previous study has demonstrated that intracerebroventricular saline administration does not impair monkey’s motor ability (Li et al., 2015), no control group receiving intracerebroventricular saline administration had been designed in this study. Before any MPTP administration, each monkey in group II received surgery and had a silicon tube inserted into the lateral cerebral ventricle. This tube acted as a guide for intracerebroventricular MPTP administration.

Before surgery, the monkeys in group II underwent a fast for 12 h. For surgery, each monkey was initially anesthetized with ketamine (10 mg/kg, i.m.) and then maintained on sodium pentobarbital (15 mg/kg, i.m.) anesthesia for the duration of the surgery. Atropine (0.1 ml/kg, i.m.) was administered with the initial ketamine injection to decrease respiratory secretions. The coordinates of the silicon tube locations were obtained through an MRI-based localization method developed by our laboratory (Jing et al., 2010). The operation was carried out according to the MRI coordinates using a stereotaxic apparatus (SN-2N, Narishige, Japan). Then the silicon tube was fixed onto the top of the skull with dental cement. The following experiments (MPTP treatments and CPP tests) were performed only after the animals had fully recovered, which usually took 7–10 days.

### Experimental Schedule

**Figure 1:**
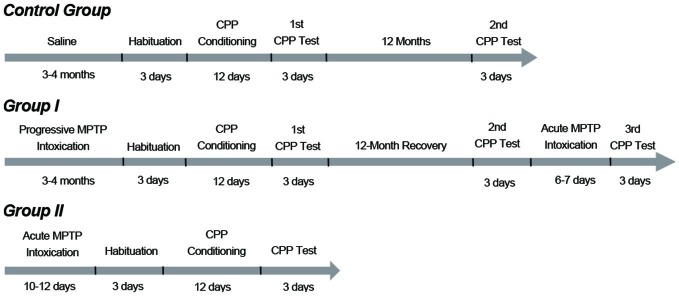
**Timelines and protocols of methyl-4-phenyl-1,2,3,6-tetrahydropyridine (MPTP) intoxication and conditioned place preference (CPP) training**. Group I monkeys initially received progressive MPTP injections for 3 to 4 months (0.25 mg/kg, i.m., injections spaced by 2–3 days) until they developed mild PD symptoms. After the progressive intoxication, the group I monkeys were subjected to the CPP procedure. The group I monkeys were tested two times in 12 months. Then these animals received acute injections of MPTP (0.45 mg/kg, i.m., 6–7 daily injections) and developed strong PD symptoms. Then the third CPP test was carried out. The group II monkeys received an acute MPTP intoxication protocol (intracerebroventricular, 5 mg per kg of brain weight, 10–12 daily injections) and developed strong PD symptoms. These animals were tested only once to examine whether they had established a morphine CPP with severe Parkinsonian symptoms. The control group performed the CPP test twice: the first one coincided with CPP training and testing of the group I monkeys; the second test was performed 12 months after the first one.

After the group II monkeys had fully recovered from the surgeries, animals in group I and group II were subjected to MPTP (Sigma, St Louis, MO, USA) treatments. The five monkeys in group I initially underwent a progressive intoxication (0.25 mg/kg, i.m., injections spaced by 2–3 days; Figure 1). Injections were repeated until mild Parkinsonian symptoms had been developed (see “Behavioral Analysis” Section described later). After the progressive intoxication, the group I monkeys were subjected to the CPP procedure (described below) and were tested twice in 12 months (Figure 1). Then these animals received an acute intoxication of MPTP (0.45 mg/kg, i.m., 6 or 7 daily injections) to damage any residual dopaminergic neurons and produce a strong symptomatic state (Figure 1). After the acute intoxication, these monkeys were retested for CPP a third time.

The three monkeys in group II received 10–12 daily intracerebroventricular injections (5 mg per kg of brain weight (Sullivan et al., 1997) to develop strong and stable symptoms (Figure 1). After the acute MPTP intoxication, the group II monkeys underwent the CPP procedure only once (Figure 1). The control group was administered saline of the same volume/mass as the MPTP-administered monkeys in group I. These animals were subjected to the CPP experiment on the day group I monkeys started the CPP experiment, and were tested twice in 12 months (Figure 1).

### Behavioral Analysis—Parkinsonian Symptoms

Infrared cameras were used to record the animals’ behavior for the analysis of Parkinsonism. The severity of Parkinsonism was evaluated using the rating scale proposed by Kurlan (Smith et al., 1993). The scale includes seven items rated between 0 and 2 or 4, with a total score of 20. It takes into account classical motor symptoms (tremor, bradykinesia, posture and arm posture) as well as other activities (arm movements, balance and defensive reaction).

Each monkey was recorded daily for 1 h (10–11 am) to evaluate the development of the Parkinsonian symptoms. Each 1 h recording was split equally into four parts (15 min each) and the scores of the four parts were averaged to obtain the daily total score. In this study, group I monkeys first developed mild Parkinsonian symptoms (scores were between 5 and 7), and then the symptoms were aggravated to establish severely impaired DA systems (scores exceeding 10) by acutely administering more MPTP after a 12-month recovery period. Group II monkeys developed strong and stable symptoms (scores exceeding 10).

### Behavioral Analysis—CPP

Prior to all CPP experiments, the monkeys received a primate collar under ketamine hydrochloride (5 mg/kg, i.m.) anesthesia at least 1 week before the start of the CPP experiment. This enabled the experimenter to take the animals to the conditioning room. The CPP experiment was conducted using the same apparatus previously described in our laboratory (Wang et al., 2012). Briefly, the conditioning space was composed of three square rooms connected in a row. The sizes of the two side rooms were 190 × 190 × 265 cm (l × w × h), respectively, and the middle room (start room) was 210 × 210 × 265 cm. Specifically colored pieces of paper were glued on the walls of the three rooms to provide different visual cues to the animals. Cameras were installed on the top of the rooms to record the monkeys’ behavior.

The CPP training procedure included 3 days of habituation, 12 days of conditioning and 3 days of CPP testing. In the adaptive phase, the 12 monkeys were individually guided into the start room where they could freely move in the three rooms for 50 min on three continuous days and thus they became completely familiar with the three rooms. After the 3 days of habituation, each animal had a preferred room that served as its saline-paired room during the conditioning, while the other side room that the monkey did not prefer served as its morphine-paired room. In the conditioning days, animals received a morphine (or saline) injection in their own cages and were subsequently taken to the morphine-paired room (or the saline-paired room) and stayed for 50 min. The dose of morphine started at 1.5 mg/kg (i.m.) and increased to 3 and 4.5 mg/kg on the two following alternate days. The highest dose of 4.5 mg/kg was then maintained for the last 3 days (Wang et al., 2012). Saline was given at the same volume as morphine on the 6 days that alternated with the morphine days. After the days of habituation and conditioning, the animals were tested for CPP. Each testing lasted for 30 min on three continuous days. Group I was tested three times: 24 h after the CPP conditioning was completed, when the monkeys maintained mild Parkinsonian symptoms (0-month CPP), 12 months later when the monkeys had recovered from the progressive MPTP treatments (12-month CPP), and 12.25 months later when the monkeys had received additional acute MPTP injections and were in a strong PD symptomatic state (12.25-month CPP). Group II were tested only one time (0-month CPP). Control group was tested at time point 0 (0-month CPP) and 12 months later (12-month CPP).

The morphine hydrochloride (C_17_H_19_NO_3_•HCl•3H_2_O) was purchased from Sheng Yang, 1st Medical Company (Sheng Yang, China).

All 12 monkeys were sacrificed at the end of the CPP experiments for histological analysis.

### Histological Analysis

After the CPP experiments, all animals received deep anesthesia with an overdose of sodium pentobarbital (40 mg/kg, i.m.) and were then transcardially perfused with saline followed by a fixative containing 4% paraformaldehyde in a phosphate buffer (0.1 M, pH 7.4). Brains were sliced in 50 μm-thick coronal sections using a freezing microtome (Leica CM1850, Germany). Ten regularly spaced sections covering the anteroposterior extent of the substantia nigra (SN) and the VTA, from each of the 12 monkeys, were processed for light microscopic analysis of tyrosine hydroxylase (TH) immunolabeling.

Immunoreactivity for TH was localized using the protocol of Mazloom and Smith (2006). Briefly, sections were stained with primary rabbit polyclonal TH antibodies (1/1000 dilution; Chemicon Intl, Temecula, CA, USA; catalog #AB152#), followed by a secondary biotinylated goat anti-rabbit immunoglobulin G (1/200 dilution; Maxim Co. Ltd., China). Visualization was achieved using an avidin-biotin-peroxidase complex (ABC standard kit; 1:200 dilution in phosphate-buffered saline; Maxim) and 3,3′-diaminobenzidine tetrahydrochloride (5%; Maxim).

Sections were examined with an Olympus CX41 microscope (Tokyo, Japan), and images were acquired with a CCD camera (Sony DP25, Tokyo, Japan) controlled by the Olympus cellSens software. TH-positive cells were counted in the ten regularly spaced sections with an image analysis system (Image-Pro Plus, Media Cybernetics, Shanghai, China). The sections were matched anatomically in each of the animals, verifying that the cross sections of the midbrain were similar in the controls and PD monkeys. The percentages of neuronal loss in the SN and VTA in the MPTP-treated monkeys were evaluated by comparison with the control values of intact monkeys.

### Data Analysis

**Figure 2:**
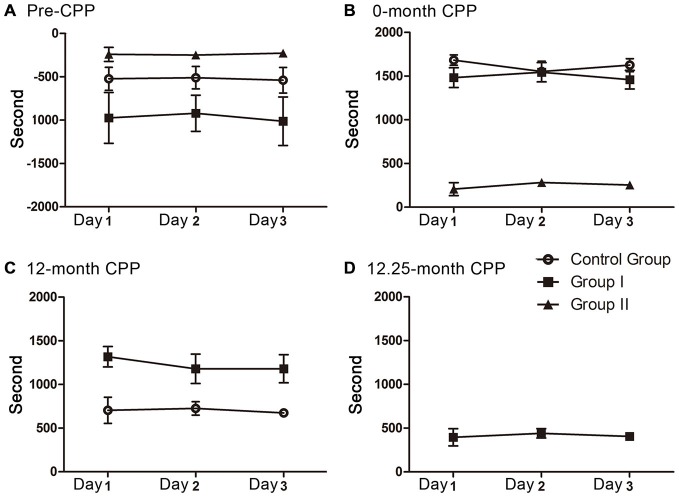
**The preference scores in the 3 days of each CPP testing**. No decay was found during each testing. **(A)** The preference scores of the three groups before CPP conditioning (*F*_(2, 9)_ = 0.012, *P* = 0.989 for control group, *F*_(2, 12)_ = 0.031, *P* = 0.970 for group I, *F*_(2, 6)_ = 0.039, *P* = 0.962 for group II). **(B)** The preference scores of the three groups during the 0 month-CPP phase (*F*_(2, 9)_ = 0.561, *P* = 0.590 for control group, *F*_(2,12)_ = 0.158, *P* = 0.855 for group I, *F*_(2, 6)_ = 0.473, *P* = 0.645 for group II). **(C)** The preference scores of group I and control group during the 12 month-CPP phase (*F*_(2, 9)_ = 0.068, *P* = 0.935 for control group, *F*_(2,12)_ = 0.283, *P* = 0.759 for group I). **(D)** The preference scores of group I during the 12.25 month-CPP phase (*F*_(2, 12)_ = 0.118, *P* = 0.890).

The time spent in the rooms during each CPP testing was recorded for analyzing the animals’ preference for morphine. The preference score was expressed by time spent in morphine-paired room minus time spent in saline-paired room (Smith et al., 2012). All 3 days of each testing were scored and then tested to find out whether the scores decay over the 3 days. One-way analysis of variance followed by Tukey’s test as *post hoc* analysis was used and no decay was found (Figure 2). Therefore, the scores of the 3 days were averaged to obtain the preference score. The walking distance was recorded as an index of locomotor activity (Wang et al., 2012).

The differences in the preference scores before and after CPP conditioning were assessed for each group using a paired *t*-test (*T*_p_) or one-way analysis of variance followed by Tukey’s test as *post hoc* analysis. The preference scores among the three groups before and after CPP conditioning were analyzed using an independent t-test (*T*_i_) or one-way analysis of variance followed by Tukey’s test as *post hoc* analysis. The walking distance among the three rooms in each group and the total walking distance among the three groups were analyzed using one-way analysis of variance followed by Tukey’s test as *post hoc* analysis.

Optical quantification of neurons in the SN and VTA were analyzed using the Mann-Whitney U test to compare group I, group II and the control group.

Differences were considered statistically significant at *p* < 0.05. All data were presented as the mean ± standard error of mean.

## Results

### Parkinsonism Aspects

**Figure 3:**
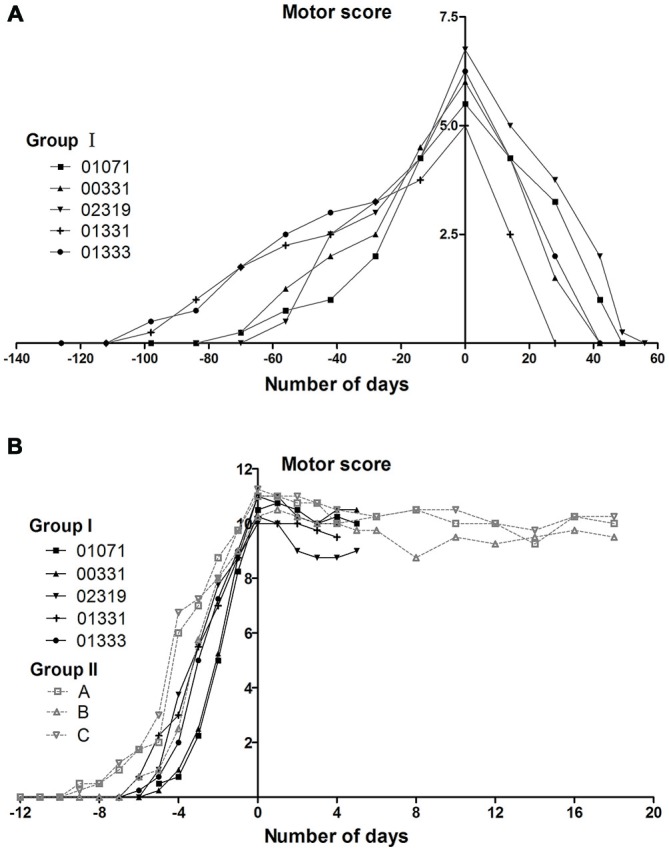
**Evolution of motor scores in group I and II monkeys during MPTP intoxication periods. (A)** The appearance and disappearance of symptoms in group I monkeys during and after the progressive MPTP intoxication. The group I monkeys received progressive intoxication and developed mild Parkinsonian symptoms (Kurlan scores between 5 and 7). Timelines are aligned such that day 0 corresponds to the day on which the maximal motor Kurlan score was obtained from an individual animal and MPTP injections were stopped. **(B)** The reappearance of symptoms in group I monkeys and the appearance of symptoms in group II monkeys during and after acute MPTP intoxications. Group I and II, which received acute MPTP intoxications, displayed severe Parkinsonian symptoms (Kurlan scores exceeding 10) and exhibited little recovery until the time of being sacrificed for histological examinations. Day 0 corresponds to the day on which the injections were stopped. The animals in group I were sacrificed on day 4 or day 5 (the day after their third CPP test). Group I monkeys are represented with continuous lines and group II with dotted lines.

The monkeys in group I initially received 12–15 (mean 13.2 ± 1.1) weeks of progressive MPTP injections for a cumulative dose of 6–7.5 mg/kg (mean 6.6 ± 0.6 mg/kg). These animals developed mild symptoms (weak tremor or bradykinesia; Kurlan scores were between 5 and 7). All of these animals experienced a total recovery after stopping MPTP treatment (Figure 3A). After receiving additional acute MPTP intoxication (6–7 days of individual injections), these animals rapidly developed severe symptoms with Kurlan scores larger than 10 (Figure 3B). Group II monkeys displayed similar Parkinsonian symptoms after their acute MPTP intoxication (10–12 days of individual injections, a cumulative dose of 5–6 mg; Figure 3B). Monkeys (in both group I and group II) that developed severe symptoms exhibited little recovery after the cessation of the acute MPTP intoxication (Figure 3B).

### CPP Aspects

#### Pre-CPP

After moving about the three rooms, all 12 of the monkeys preferred to stay a longer time in one of the three rooms in the habituation stage before morphine administration (Table 1). Furthermore, the preference scores were not statistically different among the three groups (*F*_(2, 9)_ = 3.192, *P* = 0.09). This provided a platform for the later post-CPP (0-month CPP, 12-month CPP, 12.25-month CPP) comparisons among the three groups.

**Table 1:** **The time spent in the three rooms in all monkeys during the pre-CPP phase**.

	Room 1	Room 2	Room 3
Control Group
Monkey 1	374.3	824.3	601.3
Monkey 2	192.3	847.3	760.3
Monkey 3	984.0	696.7	119.3
Monkey 4	885.0	470.7	444.3
Group I
Monkey 5	422.7	2.0	1375.3
Monkey 6	1648.0	142.0	10.0
Monkey 7	480.7	532.0	787.3
Monkey 8	175.7	24.0	1600.3
Monkey 9	137.7	998.0	664.3
Group II
Monkey 10	374.7	1264.7	160.7
Monkey 11	251.0	1464.3	83.7
Monkey 12	789.3	558.0	452.7

*Room 1 and room 3 were the two lateral rooms which served as drug-paired room or saline-paired room during the conditioning phase. The middle room (room 2) serves as the start room. Each score (seconds) in the table was obtained from averaging the three scores of the habituation days*.

#### 0-Month CPP

24 h after the morphine conditioning (0-month CPP), morphine treatment produced a significant switch in the time spent in the morphine-paired room (*T*_p_ = −18.220, *P* = 0.000 for the control group; *T*_p_ = −8.444, *P* = 0.001 for group I; *T*_p_ = −7.655, *P* = 0.019 for group II).

**Figure 4:**
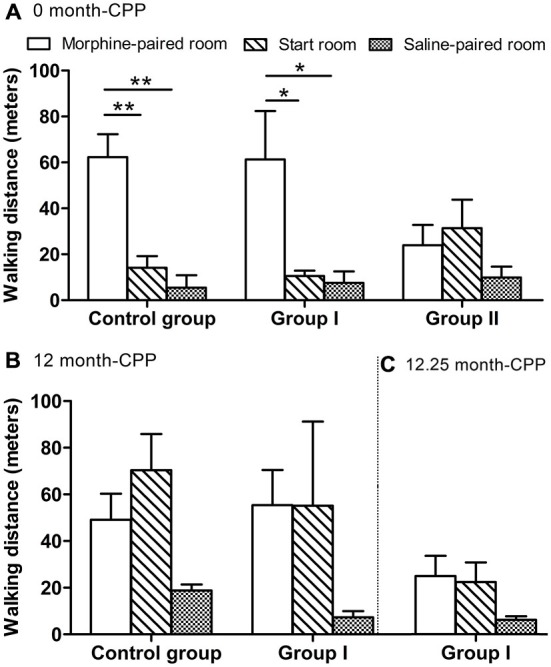
**The walking distance of all monkeys measured within a 30-min test. (A)** The walking distance in the three rooms 24 h after CPP (0 month-CPP). **(B)** The walking distance in the three rooms 12 months after CPP (12 month-CPP). **(C)** The walking distance in the three rooms 12.25 months after CPP (12.25 month-CPP). *p < 0.05, **p < 0.01. Data are presented as mean ± SEM.

During this phase, the monkeys also walked a longer distance in the morphine-paired room than in the other two rooms (*F*_(2, 9)_ = 18.106, *P* = 0.001 for control group; *F*_(2, 12)_ = 5.761, *P* = 0.018 for group I; Figure 4A). In the control group, *post hoc* analysis (Tukey’s test) showed that the walking distance in the morphine-paired room was higher than in the saline-paired room (*T* = 5.585, *P* = 0.001; Figure 4A). In group I monkeys, *post hoc* analysis (Tukey’s test) showed that the walking distance in the morphine-paired room was higher than in the saline-paired room (*T* = 3.019, *P* = 0.027; Figure 4A).

**Figure 5:**
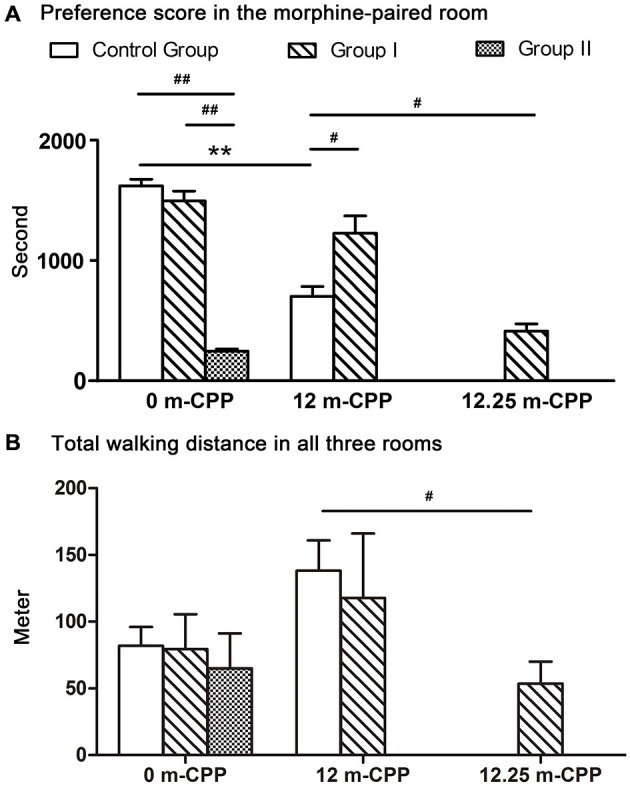
**The preference scores and the total walking distance of all monkeys measured within a 30-min test. (A)** The preference scores of time spent in the morphine-paired room after CPP conditioning. Comparisons within each group revealed that all three groups showed significant switch in their time spent in the morphine-paired room after conditioning. Comparisons among groups showed that the preference scores in monkeys with severe Parkinsonian symptoms (group I in the 12.25-month phase and group II in the 0-month phase) were significantly lower than the control group. **(B)** The total walking distance of the three rooms. Comparisons within groups showed no significant difference among different testing phases. Comparison among groups showed only the walking distance of group I at 12.25-month CPP testing phase was significantly less than the control group at 12-month CPP testing timepoint. **p < 0.01: inter-group comparisons. ^#^*p* < 0.05, ^##^*p* < 0.01: comparisons among groups. Data are presented as mean ± SEM.

The results of the comparisons among the three groups showed that there were significant differences among the three groups (*F*_(2, 9)_ = 102.770, *P* = 0.000; Figure 5A). *Post hoc* analysis (Tukey’s test) showed that the establishment of the morphine CPP in the group II monkeys was not as strong as in the other two groups (*T* = 13.122, *P* = 0.000 for controls; *T* = 12.472, *P* = 0.000 for group I, compared with group II). Conversely, the group I monkeys showed a similar preference to the control group (*T* = 1.362, *P* = 0.399; Figure 5A). The total walking distance were not statistically different among the three groups (*F*_(2, 9)_ = 0.120, *P* = 0.889; Figure 5B).

#### 12-Month CPP

Twelve months after the original morphine CPP conditioning, the monkeys in group I and the control group still prefer the morphine-paired room (one-way ANOVA between pre-CPP, 0-month CPP, and 12-month CPP, *F*_(2, 9)_ = 143.408, *P* = 0.000 for the control group, *F*_(2,12)_ = 59.620, *P* = 0.000 for group I). In the control group, *post hoc* analysis (Tukey’s test) showed that the preference score in the 12-month phase was higher than the score in the pre-CPP phase (*T* = 9.652, *P* = 0.000) and was lower than the score in the 0-month phase (*T* = 7.226, *P* = 0.000; Figure 5A). In group I monkeys, *post hoc* analysis (Tukey’s test) showed that the preference score in the 12-month phase was higher than the score in the pre-CPP phase (*T* = 8.865, *P* = 0.000) and was not statistically different from the score in the 0-month phase (*T* = 1.089, *P* = 0.538; Figure 5A). On the other hand, *post hoc* analysis showed that no difference of walking distance was found between morphine-paired room and saline-paired room (*T* = 1.932, *P* = 0.185 for control group, *T* = 1.504, *P* = 0.324 for group I; Figure 4B).

When comparing between group I and control group, monkeys in group I showed even higher preference score than control group (*T*_i_ = −2.589, *P* = 0.041; Figure 5A). The total walking distance were not statistically different between the two groups (*T*_i_ = 0.352, *P* = 0.735; Figure 5B).

#### 12.25-Month CPP

The 12.25-month test was carried out only 6–7 days after the 12-month test in group I. Because the performance did not decay over the 3 days of each testing, we assumed the performance of control group did not decay 6–7 days after the 12-month test. Therefore, the control group did not go through the 12.25-month CPP test. The preference score of group I during the 12.25-month CPP test (after the acute MPTP intoxication) was compared with the score during the 12-month test in control group. Significantly decrease was found in group I monkeys (*T*_i_ = −3.383, *P* = 0.012; Figure 5A). The total walking distance of group I was also less than the control group (*T*_i_ = 3.094, *P* = 0.017; Figure 5B). On the other hand, the walking distance in the morphine-paried room was not significantly different from the other two rooms (*F* = 2.127, *P* = 0.162; Figure 4C).

### Immunohistochemical Aspects

**Figure 6:**
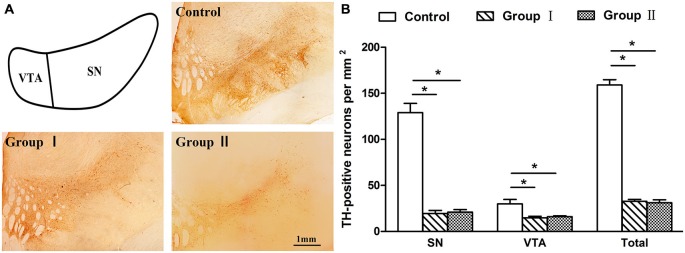
**Tyrosine hydroxylase (TH) labeling in the mesencephalon of the rhesus monkeys. (A)** Depiction of areas used to define the SN and ventral tegmental area (VTA) and examples of the distribution of TH immunostaining in the mesencephalon from the control group, group I and group II, respectively. Scale bar: 1 mm. **(B)** Density of TH-positive neurons that remained in the mesencephalon of three groups. *p < 0.05. Data are presented as mean ± SEM.

An immunohistochemical analysis of TH was performed on all groups to confirm that the loss of dopaminergic neurons in the group I and II monkeys that had severe Parkinsonian symptoms at the time of sacrifice was similar to or larger than the 70% loss of dopaminergic neurons witnessed in previous studies (Burns et al., 1983; Elsworth et al., 2000; Mounayar et al., 2007). The monkeys in group I and group II both exhibited an extensive loss of TH-positive neurons in comparison to the control monkeys evaluated in this study (group I: 79%, *P* = 0.011; group II: 80%, *P* = 0.019; Figure 6). In the VTA, the loss of TH-positive neurons in group I and group II was 50 and 47%, respectively. The SN was more sensitive with a loss of 85% in group I and a loss of 84% in group II. In addition, no statistical difference in cell loss was found between the group I and group II monkeys (*P* = 0.881 for SN and VTA; *P* = 0.655 for total).

## Discussion

Reward has been associated with dopaminergic function. The results of this study, the first to be carried out on rhesus monkeys, found that severe dopaminergic neurons loss impaired CPP for morphine. This study confirmed previous studies carried out on rodents that had suggested that DA had the ability to facilitate reward related behaviors (Berridge and Robinson, 1998; Cannon and Palmiter, 2003; Hnasko et al., 2005). Moreover, this study also revealed that this ability of DA had not been impaired in monkeys existing mild Parkinsonian symptoms as these monkeys with can normally establish and retain a morphine preference. The previous rodent studies had not been able to make this point as the rodents were in a severe state and had less than 1% of normal brain DA concentrations when the previous preference experiments were performed (Berridge and Robinson, 1998; Hnasko et al., 2005).

According to previous studies, reward contains three components: liking, learning and wanting (Berridge and Robinson, 1998; Berridge, 2007). The association of the sensory pleasure (liking) of morphine with the environment (dependent on learning) is formed during the CPP conditioning session (CPP acquisition), and then is revealed during the testing phase (Hnasko et al., 2005). Wanting is a motivation response triggered by and assigned to a reward-related stimulus and is evident during the testing phase (CPP expression). Without motivation, the animal may simply sit still and not manifest a preference (Berridge, 2007). In this study, group I monkeys with mild Parkinsonian symptoms displayed a strong preference for the morphine-paired room which was similar to the controls. This result suggested that existing mild Parkinsonian symptoms would not affect any component of reward.

Furthermore, when monkeys exhibited severe Parkinsonian symptoms, they spent less time in the morphine-paired room than did the controls (Caution must be exercised in interpreting the results from Group I at the 12.25 month timepoint as we did not perform a CPP test at the 12.25 month timepoint in the control group, and as such the reduced CPP score could result from the repeated testing). Thus, severe loss of dopaminergic neurons provided profound constraints on CPP performance. Because these monkeys still had the motor ability and readily explored the CPP rooms, motor deficits failed to explain the less time monkeys spent in the morphine-paired room after the development of severe Parkinsonian symptoms. However, the results from the group I monkeys suggested that this abnormal performance may contribute to the deficit in motivation for morphine. In the group I monkeys, the preference score was initially similar to the controls (0-month and 12-month CPP tests), which meant that they had acquired CPP normally. Therefore, the significantly decreased score at the 12.25 month timepoint provided evidence that severe DA depletion reduced animals’ wanting for morphine.

In this study, only group II monkeys acquired CPP with severe dopaminergic neuron loss. However, the significantly lower score in group II (compared to control group) during the 0-month testing phase failed to reveal whether severe DA depletion impaired the acquisition of CPP, as the CPP testing phase included not only the acquisition of CPP but also wanting, and group II monkeys’ wanting for morphine was possibly impaired (inferred from the evidence of the 12.25-month test in group I). Combining with the result of the group I (0-month testing phase), our data only proved that mild Parkinsonian symptoms would not affect monkey’s acquisition of CPP.

As demonstrated in this DA-deficient primate model, DA has the ability to facilitate the manifestation of place preference. Furthermore, this ability only begins to become impaired when the degree of Parkinsonian symptoms exceeds a mild state. This novel finding, obtained from monkeys, provides a good basis for further investigations into the neural pathways and brain structures (e.g., the medial prefrontal cortex) involved in reward development.

## Author Contributions

TY and YM conceived and designed the experiments. TY, JW, SY performed the experiments. TY, JW analyzed the data. XH contributed reagents/materials/analysis tools. TY, JDR wrote the paper.

## Conflict of Interest Statement

The authors declare that the research was conducted in the absence of any commercial or financial relationships that could be construed as a potential conflict of interest.
